# Use of Healthy Emulsion Hydrogels to Improve the Quality of Pork Burgers

**DOI:** 10.3390/foods11040596

**Published:** 2022-02-18

**Authors:** Danila Foggiaro, Rubén Domínguez, Mirian Pateiro, Aurora Cittadini, Paulo E. S. Munekata, Paulo C. B. Campagnol, Maria João Fraqueza, Pasquale De Palo, José M. Lorenzo

**Affiliations:** 1 Department of Veterinary Medicine, University of Bari A. Moro, Valenzano, 70010 Bari, Italy; d.foggiaro@studenti.uniba.it (D.F.); pasquale.depalo@uniba.it (P.D.P.); 2 Centro Tecnológico de la Carne de Galicia, Parque Tecnológico de Galicia, Avd. Galicia n° 4, San Cibrao das Viñas, 32900 Ourense, Spain; rubendominguez@ceteca.net (R.D.); mirianpateiro@ceteca.net (M.P.); aurora.cittadini@unavarra.es (A.C.); paulosichetti@ceteca.net (P.E.S.M.); 3 Instituto de Innovación y Sostenibilidad en la Cadena Agroalimentaria (IS-FOOD), Universidad Pública de Navarra (UPNA), Arrosadia Campus, 31006 Pamplona, Spain; 4 Departmento de Tecnologia e Ciência de Alimentos, Universidade Federal de Santa Maria, 97105-900 Santa Maria, Brazil; paulocampagnol@gmail.com; 5 CIISA—Centro de Investigação Interdisciplinar em Sanidade Animal, Faculdade de Medicina Veterinária, Universidade de Lisboa, Avenida da Universidade Técnica, 1300-477 Lisboa, Portugal; mjoaofraqueza@fmv.ulisboa.pt; 6 Universidade de Vigo, Área de Tecnoloxía dos Alimentos, Facultade de Ciencias, 32004 Ourense, Spain

**Keywords:** healthy burgers, fat replacement, fatty acid profile, sensory analysis

## Abstract

The present research evaluated the use of oil mixture emulsion hydrogels as animal fat replacers and their effect on the physicochemical, nutritional and sensory characteristics of pork burgers. Three different types of burgers were manufactured: control (samples elaborated with 100% pork fat), T1 and T2 (pork fat totally replaced by emulsion hydrogels of walnut or pistachio oil and algal oil, respectively). Fat replacement increased the moisture and ash contents and colour parameters (L* and b*) of pork burgers. Modified samples turned out to be firmer and chewier than those in the control group. The addition of oil emulsion hydrogels caused a significant decrease in fat and energy contents and the products obtained can be considered “reduced fat content”. Moreover, the content of saturated fatty acids decreased, while mono- and polyunsaturated fatty acids increased, constituting an improvement in health indices. Sensory differences were found between the samples and T2 was the most preferred for flavour and overall. However, both modified burgers had good levels of acceptability. To conclude, the use of the proposed oil mixture emulsion hydrogels as pork backfat substitutes represents a promising strategy to obtain healthier pork burgers without negatively affecting technological or sensory properties.

## 1. Introduction

Although improvements in living conditions are observed, diet-related chronic diseases have become more common [1]. Meat is a fundamental component of the Western diet [2] and is important for human health and a balanced diet because it is a source of essential nutrients and compounds of high biological value [3,4]. However, in recent years meat products have been the focus of awareness campaigns, mainly due to their high contents of saturated fatty acids (SFA) and cholesterol, which are associated with several chronic diseases [5,6,7]. Burgers are one of the most consumed meat products in several countries due to their sensory characteristics, low price, convenience and fast preparation [8,9], but also due to the current increase in the number of fast foods [10]. However, this product is not recommended for a healthy diet [9] since it contains a high amount of animal fat in its formulation (from 9% to 20%) with high SFA content (31–42%) [5,6,9,11,12]. In fact, it is well known that the excessive consumption of fats, especially SFA, can increase the risk of certain diseases [13]. Thus, to reduce the incidence of such diseases, food-based dietary guidelines from several international health organizations (WHO and EFSA) have issued recommendations on the intake of total, saturated and trans fats [14] and on the limit of consumption of processed meats [15,16]. Nevertheless, a large number of consumers are becoming increasingly aware of the health implications of frequent consumption of animal fats. This awareness has changed the population’s diet, causing a growth in the demand for low-fat and healthier products [17,18]. Most consumers do not accept “imitation meat” or “knitted steaks” [2,19] since they prefer meat with better nutritional quality, of course without sacrificing the sensory quality of foods. This has forced the meat industry to undertake the reformulation of some traditional preparations [20,21], and the challenge for research and the meat industry is to find valid alternatives to improve the lipid profile in meat products without negatively affecting their technological and sensory qualities [3,9].

In the literature there are numerous studies that have tried to improve the nutritional value of meat products by reducing or replacing ingredients associated with health risks, mainly fats [22]. Animal fat has been replaced by plant oils, marine oils [7,23], grounded plant seeds [24], cereal brans [25], and fruit flours [26].

The substitution of animal fat with vegetable and/or marine oils to make healthier meat products has appeared as one of the most promising means of improving the nutritional quality of meat products [9,21,23,27]. Several studies can show that this approach is useful to increase monounsaturated (MUFA) and polyunsaturated fatty acids (PUFA) and reduce SFA contents [5,7,9,10,12,21,23,28,29]. On the other hand, using n-3 PUFA-rich oils allows for a reduction in the n-6/n-3 ratio in modified products and to claim that a product is a source “a source of omega-3” [30]. However, the simple incorporation of different fat sources could have several adverse effects on the product, mainly in terms of technical and sensory properties, resulting in lower acceptance [3,6,14,31]. This is because animal fat plays an essential role in the quality of meat products [9,12,32,33]. In order to minimize the possible negative consequences in terms of product quality, different strategies have been suggested to structure liquid oils, obtaining reformulated lipid systems with similar properties to animal fat [14,34]. The techniques proposed include oil encapsulation [34] and oil structuration [14].

Multiple researchers have evaluated different techniques for converting liquid healthy oils into a solid-like gel [14], highlighting mainly two strategies: the production of oleogels [35] and emulsion hydrogels [5,7,11,21,23,34]. Oleogels are mainly composed by oil (>90%), which is gelled with organogelators [14], while an oil emulsion hydrogel is a semi-solid system in which oil (disperse phase) and water (continuous phase) are immobilized in a three-dimensional network structure made by gelling agents [36,37,38]. In a recent review published by Dominguez et al. [34], it is pointed out that emulsion hydrogels for the replacement of animal fat present several advantages in comparison with oleogels. Moreover, the use of low temperatures during hydrogel manufacture makes it much less aggressive with respect to thermolabile substances in oils [5,14]. Additionally, the use of oil mixture instead of pure oil allows the optimization of the nutritional value of the meat product, improving the fatty acid profile and minimizing the technological and sensory effects [34]. Several ingredients have been used to realize this kind of hydrogel emulsion [39]. However, among the studied techniques, the use of alginate gel has shown excellent results [5,23], and several recent studies have analysed the use of this formulation with different vegetable/marine oils [5,7,21,23,40].

Algal oil is an excellent source of long-chain omega-3 fatty acids, such as eicosapentaenoic (EPA, C20:5 n-3) and docosahexaenoic (DHA, C22:6 n-3) acids [12]. These fatty acids had several health benefits. Thus, the use of algal oil for the improvement of meat products’ nutritional quality has been proposed [23]. On the other hand, oils obtained from nuts (walnuts, pistachios and almonds) also have potential nutritional properties [41].

Observational studies have shown that nut intake has beneficial impacts on human health [42,43]. Walnuts have one of the highest oil contents among nuts [44] and are rich in linoleic acid and therefore in PUFA [45,46,47]. They also have a good n-6/n-3 ratio, which helps to prevent diseases [48]. Pistachio oil is a source of monounsaturated fatty acids (MUFAs), mainly oleic acid [49,50,51], with peculiar and pleasant sensory characteristics [41]. Among meat products, due to their high fat content and popularity, burgers represent an attractive choice for the production of fat-reduced products [6]. Therefore, taking into account the previous results and the organoleptic characteristics of the aforementioned oils, the aim of the present study was to improve the nutritional value of pork burgers through the total replacement of pork backfat with two different oil alginate-based emulsion hydrogels (formulated with a mixture of algal oil and pistachio or walnut oil) and to evaluate how this modification affects the physicochemical, nutritional and sensory characteristics.

## 2. Materials and Methods

### 2.1. Oil Emulsion Hydrogels Preparation

All stages of the processing were performed in the Centro Tecnolóxico da Carne (CTC) San Cibrao das Viñas, Ourense, Spain. The present study involved the preparation of two different alginate-based emulsion hydrogels: treatment T1, containing 35.05 g/100 g of walnut oil and 2.25 g/100 g of algal oil; and treatment T2, containing 35.05 g/100 g of pistachio oil and 2.25 g/100 g of algal oil. Pistachio oil (Tenuta del Roero, F.lli Ruata S.p.a., Baldissero d’Alba, Italy) and walnut oil (Naturgreen, Murcia, Spain) were sourced from a local market. The algal oil came from the Solutex Corporation (Madrid, Spain) and it has been used due to its high omega-3 amounts. The fatty acid profiles of fat/oil sources (analyses performed in triplicate) are shown in Table 1. The determination of fatty acid profiles was carried out according to the protocol described by Barros et al. [5].

The oil emulsions were prepared following the steps applied by Cittadini et al. [7]. Prosella powder is a commercial mixture composed of jellifying agents (calcium sulphate and sodium alginate), wheat glucose syrup (7.4%), a stabiliser (disodium diphosphate, added P_2_O_5_: 9.58%) and an antioxidant (sodium ascorbate), according to information provided by the manufacturer. The hydrogel was prepared by mixing water (56 g/100 g, pH 7.7 at 10 °C) with algal and walnut or pistachio oil in a bowl cutter (Sirman, modC15VV, Marsango, Italy) for 1 min. Subsequently, Prosella powder (6.7 g/100 g) was added to the bowl cutter and the mixture was homogenised for 3 min. The mixture was transferred to a container and left to rest for 2 h. Then, the hydrogel was kept at 4 °C until the production of the burgers. The final proportion of oils in the hydrogels was 37.3 g/100 g [7].

### 2.2. Pork Burger Formulation and Processing

**Figure 1:**
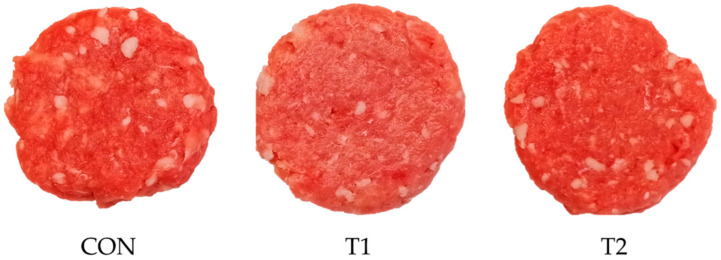
Appearance of pork burgers. CON: control burgers; T1: burgers formulated with walnut and algal oil mixture hydrogel; T2: burgers formulated with pistachio and algal oil mixture hydrogel.

Three different treatments were processed: CON (control), containing 10 g/100 g of pork back fat; and two other reformulations containing oil emulsion hydrogels as total animal fat replacers: T1, containing the hydrogel emulsion with walnut oil mixed with algal oil; and T2, containing pistachio and algal oils, both in 10 g/100 g (Figure 1). All formulations also included the following common ingredients: lean pork meat (82 g/100 g) (provided by Cárnicas M.BOO S.L., Ourense, Spain), salt (1.05 g/100 g) and water (7 g/100 g). The pork burgers were manufactured according to Barros et al.’s [5] procedure. Five replicates were produced for each formulation and the same elaboration was replicated three times, on different days. After processing, proximate composition, physicochemical parameters, fatty acids profile and sensory characteristics of the burgers from each treatment were analysed [23].

### 2.3. Physicochemical Analysis

#### 2.3.1. Chemical Composition and Caloric Value

The moisture, protein and ash contents were determined following the protocols defined by the ISO Standard methods [52,53,54]. In the case of total fat content, the official procedure Am 5-04 from the American Oil Chemists’ Society was used [55]. The caloric value was calculated according to European Commission regulations [56].

#### 2.3.2. pH and Colour

The pH of the samples was measured with a digital portable pH meter using a penetration probe (Hanna Instruments, Eibar, Spain). The colour of the burgers was determined in the CIELAB space (L*, a* and b* represent lightness, redness and yellowness, respectively) with a colorimeter (Konica Minolta CM-600d, Osaka, Japan). The equipment has an 8 mm aperture size with 0° viewing angle geometry and a pulsed xenon arc lamp (illuminant D65). The instrument was calibrated using a white ceramic tile prior to analysis. Three random points in each sample were for colour determination.

#### 2.3.3. Cooking Loss and Texture Profile Analysis

The burgers were vacuum packaged and cooked in a water bath (JP Selecta, Precisdg, Barcelona, Spain) with automatic temperature control until reaching internal temperature of 70 °C. Heating was monitored with a tyke-K thermocouple (comark, PK23M, St Neots, UK) wired to a data logger (Comark Dilligence EVG, N3014). Once cooked, the samples were allowed to cool for 30 min at room temperature (22 °C).

The methods described by Echegaray et al. [57] were used to determine the cooking loss and for the texture profile analysis. Briefly, cooking loss was calculated as the difference between the weights of cooked and raw burgers. The texture profile analysis was determined using a flat surface cylindrical probe (19.85 cm^2^) at room temperature until reaching 60% compression. The force time curves were registered with a cross head speed of 3.33 mm/s. The Texture Exponent 32 software (version 1.0.0.68, StableMicro Systems, Vienna Court, UK) was employed to determine hardness (N), springiness (mm), cohesiveness, gumminess (N), and chewiness (N·mm).

### 2.4. Determination of Fatty Acid Profiles and Health Indices

#### 2.4.1. Fatty Acid Profiles of Burgers and Fat Sources

Fatty acid profiles were quantified according to a previous study [5]. The extraction of total fat content was carried out using the method described by Bligh and Dyer [58] with modifications. Ten grams of fat source were thoroughly homogenised for 30 s with a mixture of 10 mL of chloroform and 20 mL of methanol. Then, 10 mL of chloroform and 10 mL of 1% NaCl solution were added and homogenized for 30 s. The layer composed of chloroform and the fatty acids was separated from the aqueous layer with residue by centrifugation (10 min at 4000 rpm). The chloroform was separated from fatty acids using a N_2_ gas stream. Fatty acids were reserved for transesterification.

Transesterification was carried out following the method used by Domínguez et al. with modifications: 20 mg of extracted fat was dissolved in 1 mL of toluene and homogenised with 2 mL of 0.5 N sodium methoxide solution. Then, the mixtures were vortexed for 10 s and rested for 15 min at room temperature. Posteriorly, 4 mL of 10% H_2_SO_4_ in methanol was added and vortexed for a few seconds. Two millilitres of saturated sodium bicarbonate solution were added and vortexed again for a few seconds. The formed fatty acid methyl esters (FAME) were extracted using 1 mL of hexane followed by 10 s of homogenisation in a vortex for a few seconds and then the organic phase was transferred to a GC vial.

The FAMEs were separated and quantified using a gas chromatograph (GC-Agilent 7890B, Agilent Technologies, Santa Clara, CA, USA) equipped with a flame ionization detector (FID) and PAL RTC-120 auto-sampler. The sample (1 μL) was injected in split mode (1:50) in the injector kept at 250 °C with a total flow of 64.2 mL/min. The DB-23 fused silica capillary column (60 m, 0.25 mm i.d., 0.25 μm film thickness; Agilent Technologies) was used to separate the FAMEs using the following conditions: initial oven temperature of 50 °C with a holding time of 1 min, followed by temperature increases of 25 °C/min until 175 °C, 4 °C/min until 230 °C with a holding time of 5 min and 4 °C/min until 240 °C with a holding time of 2.75 min. The selected carrier gas was helium with a 1.2 mL/min flow rate and 22.9 psi pressure at the column head.

The FID detector was kept at 280 °C with operational flows of H_2_ at 40 mL/min, air at 450 mL/min and makeup flow at 30 mL/min. The total time for chromatographic analysis was 30 min. The software Mass Hunter GC/MS Acquisition B.07.05.2479 (Agilent Technologies) was used to control the equipment and acquire data. Subsequent data analysis was performed in the program Mass Hunter Quantitative Analysis B.07.01. The identification of each FAME was determined by comparison of retention times with authenticated standards (FAME Mix-37 components; docosapentaenoic acid (C22:5n-3; DPA); trans-11 vaccenic acid (11t-C18:1; TVA); cis-vaccenic acid (18:1n-7, CVA, Supelco, Madrid, Spain) and conjugated linoleic acid (9c,11t-C18:2, CLA, Matreya, State College, PA, USA)). Results were expressed as g/100 g of total fatty acids identified.

#### 2.4.2. Health Indices of Burgers

The atherogenic (AI) and the thrombogenic (TI) indices [59], the hypocholesterolaemic/hypercholesterolaemic ratio (h/H) [60] and the n-6/n-3 and PUFA/SFA ratios were calculated.

### 2.5. Sensory Evaluation

The sensory analysis of the reformulated pork burgers was conducted at the Centro Tecnolóxico da Carne (CTC) in which 38 consumers (from both genders) from Ourense (Spain), who were usually meat consumers, participated in the test in five sessions. The number of consumers can be considered appropriate, according to Mammasse and Schlich [61]. Each consumer tasted three cooked samples following the procedure described by Vargas-Ramella et al. [21]. Briefly, the burgers were prepared in an oven equipped with a temperature probe (Rational Combi Master^®^ Plus CMP61, Landsberg am Lech, Germany) until reaching a core temperature of 70 °C. Once cooked, 2 cm^3^ samples were individually wrapped in foil, identified with arbitrary 3-digit codes, and kept in a heater (55 °C for up to 30 min). The order of sample presentation was also randomized. The consumers evaluated the acceptance of appearance, cooked odour, firmness, juiciness, fatty character, flavour, and overall liking of burgers using a seven-point hedonic scale (from score 1 “disliked very much” to score 7 “liked very much”). Water and toast were also provided to panellists. In addition, a preference test was also carried out [62] using a three-point scale (1 = least favourite and 3 = most favourite).

### 2.6. Statistical Analysis

The production of burgers (five treatments) was replicated thrice on separate days. Statistical analyses of obtained results were performed using the Statistical Analysis System (SAS version 9.4, SAS Institute Inc., Cary, NC, USA). After checking the normal distribution and variance homogeneity (by means of the Shapiro–Wilk test), data were examined using the analysis of variance (ANOVA) by setting the parameters as dependent variables, the fat source as fixed effects and the replications of the entire experiment as random effects. Duncan’s multiple range test was applied to identify the mean differences among treatments. Regarding the sensory analysis, consumers were additionally included as a random effect (each panellist tasted three samples, one for each treatment, in a single session). The statistical evaluation for the preference test was performed using the Friedmann test, with Newell and McFarlane tables (*p* < 0.05). When a significant effect (*p* < 0.05) was found, a least significant difference (LSD) test was used as a multiple comparison test [7].

## 3. Results and Discussion

### 3.1. Proximate Composition and Physicochemical Analysis

The total lipid replacement significantly affected the proximate composition and physicochemical parameters of the pork burgers. Table 2 presents the results of the chemical and physicochemical analysis.

As expected, in modified samples the moisture and ash proportions increased significantly (*p* < 0.001) compared to the control sample because of the amount of water in the gel and the alginate powder added in the treatments, respectively [23]. These outcomes agree with results reported by different authors who have studied the implementation of animal fat replacement by alginate-hydrogel emulsions with healthful oils in burgers [5,7,21,23]. The chemical analysis did not report significant differences in protein content between control and modified samples, as in other similar studies [11,29,63]. In contrast, other researchers [5,7,12,28] observed a decrease in protein content, since animal back fat consists of about 10% protein [11]. On the other hand, these products can be called “a source of protein” according to Regulation (EC) No 1924/2006 [64] since at least 20% of the energy value of the product is contributed by proteins.

In agreement with several studies, the animal fat replacement resulted in a significant (*p* < 0.001) decrease in fat content in comparison to the control group. These results reflected the low oil proportion used in the hydrogels (37.2%). Concerning fat reduction, the present study accords with other studies in which animal fats were totally or partially replaced with various healthy oils, such as walnut oil [28], tiger nut oil [5,40], avocado or pumpkin seed oils with algal oil [7], chia, tiger nut and linseed oils [11,21], canola oil [9,29,65], olive oil [63], algal oil [12] and a mixture of wheat germ and algal oils [23].

In this regard, the current Regulation (EC) No 1924/2006 [64] for nutrition claims reports that for food to be labelled as “reduced fat content” the fat amount must be reduced by at least 30% [23]. Therefore, T1 and T2 samples can be considered as “reduced fat content” because they show, respectively, a 41.07% and 43.14% fat reduction compared to the control. The energy value was also significantly reduced in the reformulated samples, with a recorded decrease of 24.46% in T1 and 26.13% in T2 compared with CON samples (*p* < 0.001). This reduction is directly related to the reduced fat content [23], which is known to represent the most important component of total energy. Similar trends were reported by Barros et al. [5,23] and Cittadini et al. [7] in studies of the incorporation of healthy oils in burgers as an animal fat substitute. Concerning colour parameters, Table 2 shows that the treatments significantly affected L* (*p* < 0.001) and b* (*p* < 0.05) values, while no effect was observed in a* values, in accordance with the observations of Barros et al. [5] and Serdaroğlu [63]. Probably the reason for the L* value increase is that the diameter of oil globules in hydrogels is smaller than the globules in animal fat such that they increase light reflection [5], while the increase in b* may be because pistachio and walnut oils are more yellowish in colour than pork back fat, which has a whitish colour (visual assessment). Regarding the results for the burgers, the use of hydrogels increased L* and b* and decreased a* [14], but in the literature we can find highly contrasting results: for instance, Barros et al. [23] observed no colour differences between control burgers and burgers made with algal and wheat germ oil emulsions instead of animal fat, Cittadini et al. [7] reported a decrease of L*, a* and b* values in foal burgers made with avocado and pumpkin seed oil hydrogels, while other researchers, such as Vargas-Ramella et al. [21], have observed an increase of a* value in deer burgers made with chia, linseed and tiger nut oil hydrogel emulsions. It is well known that several factors could be responsible for the presence of different outcomes in colour analyses, such as the colour, characteristics and composition of the oil, the colour of the gelling agents as well as the properties of all the other ingredients and the interactions between them [28].

Furthermore, our reformulation did not affect pH values, as reported by previous authors, who similarly did not find pH differences between the control group and treatments with different vegetal fat sources [7,9,21,23,65,66,67].

Cooking loss behaviour was not affected by the treatment according to Barros et al. [23] and Heck et al. [11]. However, cooking loss is normally affected by several factors [28,68] and therefore our outcomes are in disagreement with previously published studies that reported a reduction of cooking loss in modified foal [7], beef [9,23,35] and deer [21] burgers. In our case, the lipid reformulation was effective to maintain these important technological parameters [11].

Regarding TPA, the analysis showed an increase of all texture parameters except springiness, showing a similar pattern to the results obtained by Barros et al. [23] using hydrogel emulsions of chia, linseed and tiger nut oils to replace pork backfat. T1 and T2 samples showed similar values to each other and contrasted with the control sample, having a firmer texture and higher cohesiveness, gumminess and chewiness. Barros et al. [23] state that it is difficult to attribute these changes to a single factor. Therefore, according to Barros et al. [23], the outcomes do not allow us to state with certainty which effect has the greatest influence on the change in the consistency of the reformulated burgers. Nevertheless, the results obtained in this study are in disagreement with many other studies that obtained similar values for hardness [5,7,21,40], cohesiveness [5,7,21,40], chewiness and gumminess [5,21,40] in reformulated burgers. However, in other studies, a reduction in gumminess [28] and chewiness has been reported, e.g., by Cittadini et al. [7] and Serdaroğlu et al. [63].

### 3.2. Fatty Acid Profiles and Healthy Indices

#### 3.2.1. Fatty Acids

This study aimed to enhance the nutritional property of traditional pork burgers made with pork backfat. In terms of lipids, the reformulation with alginate-based emulsion hydrogels had a positive effect on the total fatty acid profile of pork burgers (Table 3). In this table, only fatty acids that represented >0.1% were presented, while all the fatty acids detected have been considered for the calculation of SFA, MUFA, PUFA, n-3, n-6 and nutritional indices [5].

In the sample made with animal fat (control) the major fraction of fatty acids was represented by MUFAs (38.43 g/100 g FA), followed by SFAs (36.12 g/100 g FA) and PUFAs (15.99 g/100 g FA), and the fatty acid with the highest concentrations was C18:1n-9, followed by C16:0, C18:2n-6 and C18:0, respectively. As expected, the lipid profile of the oils used was reflected in the T1 and T2 formulations, thus the reformulation had noticeable effects on the fatty acid contents of the treatments. In fact, the SFA amount was significantly (*p* < 0.001) reduced in modified burgers, as reported by several other studies in the literature, in which animal fat was replaced by vegetable and/or marine oils [5,7,12,21,23,28,29]. These outcomes are mainly related to the lower values of C16:0 and C18:0 obtained in the reformulated samples. Therefore, according to the European Regulation (CE) 1047/2012 [69], reformulated burgers can be claimed as “reduced saturated fat” since they have a 30% reduction in SFA compared to the original product. Moreover, following the SFA recommendations [21], the burgers produced in this experiment can certainly be considered healthier when compared with the traditional product. In line with the fatty acid profile of the employed oil, the MUFA amount increased in the T2 sample, formulated with pistachio oil, and decreased in the T1 sample, formulated with walnut oil. It is important to point out that these increases in MUFA content in burgers prepared with pistachio oil were due to the higher (*p* < 0.001) MUFA content in the starting oil compared with the contents observed in walnut oil and pork backfat (Table 1). The most prevalent monounsaturated fatty acid in all the samples was C18:1n-9, which is mostly represented in the T2 sample, with 40.30 g/100 g of fat, followed by 33.10 g/100 g of fat in the control and 26.03 g/100 g of fat in the T1 sample. Moreover, as stated by other authors [7], other individual MUFAs besides C18:1n-9, such as C16:1n-7 and C18:1n-7, were present in modest amounts in T2 burgers. As expected, the use of different oils resulted in different behaviours in the two formulations, as found in the study of Cittadini et al. [7], in which the T1 sample (prepared with 100% pork fat replaced by avocado and algal oils) was found to have the higher MUFA content due to the higher content of monounsaturated fatty acids in the oil used for the preparation (avocado oil). In addition, Vargas-Ramella et al. [21] reported a similar situation for three treatments with substitutions of animal fat by tiger nut, chia and linseed oils.

The decrease in SFAs in the treatments accompanying the increase (*p* < 0.001) in PUFAs translated into an additional beneficial effect for the modified burgers. This trend is due to the peculiar fatty acid composition of the oils employed. In particular, the T1 treatment presented a higher profile of PUFAs (Table 3) since the walnut oil had a very high C18:2n-6 and C18:3n-3 content (Table 1) compared to the pistachio oil which, as previously mentioned, was abundant in C18:1n-9 (Table 1). Overall, the T2 sample still has the highest PUFA content compared to the control group. These results are consistent with the literature, in which several other authors reported that the reformulation of meat products with vegetable and/or marine oil hydrogels causes a significant increase in unsaturated fatty acid contents [5,7,23,28,29,70,71]. As mentioned before, marine oils contain high amounts of n-3 fatty acids [12] and the results obtained in the present study can confirm this. In fact, the addition of algal oil in the elaborations increased (*p* < 0.001) omega-3 fatty acid values compared to the control group. In the T2 sample a slightly higher quantity of long-chain n-3 fatty acids can be observed, in line with the quantity present in the starting oil.

For this purpose, due to the high amount of omega-3 fatty acids (T1: 89.45 mg/100 g of product, T2: 87.99 mg/100 g) (data not shown), modified burgers can be claimed as having a “high omega-3 content” because they are within the range reported by the European Parliament Regulation (EU) No 116/2010 [30] which establishes a minimum of 80 mg of the sum of EPA and DHA per 100 g of product. Our data were confirmed by previous publications which studied the use of algal oil gelled emulsion as a substitute for animal fat, e.g., Barros et al. [23], who used seaweed oil and/or wheat germ oil in the elaboration of beef burgers, and Cittadini et al. [7], who reported that the omega-3 fatty acid content was increased in foal burgers with alginate-based emulsion hydrogels containing algal oil. The high omega-6 concentration in the burgers reformulated with walnut oil emulsion was expected, since walnut oil has a higher C18:2n-6 proportion than pistachio oil, as reflected in the n-6/n-3 index.

#### 3.2.2. Health Indices

In order to verify how the addition of pistachio and walnut oils impacts the health profile of pork burgers, some nutritional indices were calculated (Table 3). The n-6/n-3 ratio was assessed and T1 showed the lowest n-6/n-3 ratio (4.38), followed by T2 (9.10), and, as expected, the highest value was found in the control sample (13.93). As described above, this fact can be explained by the high omega-3 content, in particular, DHA, found in algal oil (Table 1). However, both T1 and T2 are in line with the recommendation of the EFSA [72] which states that the n-6/n-3 ratio should be less than 4.0 for healthier products [73]. Even so, the results derived by the n-6/n-3 ratio should not be considered alone [7]. Another considered health index is the PUFA/SFA ratio that, in keeping with the recommendations, should be above 0.4 [74]. Both T1 and T2 are in accordance with the recommended ratio, showing that the use of alginate-based emulsion hydrogels could improve the nutritional characteristics of pork burgers. In addition, among healthy indices, we find a reference to the atherogenic (AI) and thrombogenic (TI) indices, which values should be as low as possible [59]. In our study, both reformulated burgers showed lower AI and TI values compared to the control group, and the lowest value can be observed in the sample reformulated with walnut oil (T1). Lastly, hypocholesterolemic/hypercholesterolemic (h/H) indices were calculated and both treatments showed an increase, in line with the recommendations. All these results agree with those obtained by other authors who mentioned that burgers made with either partial or total animal fat replacement by hydrogel emulsions had better health indices in comparison with burgers manufactured with animal fat [7,11,21,23,28].

### 3.3. Sensory Analysis

**Figure 2:**
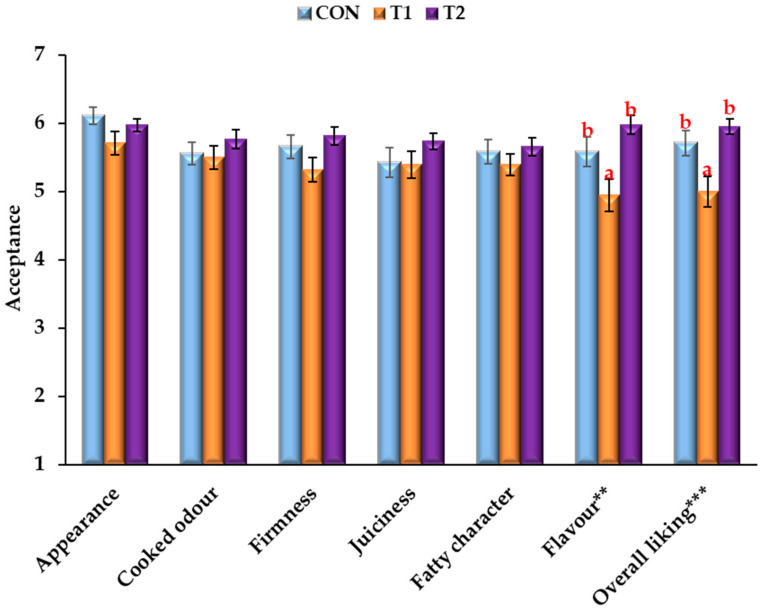
Acceptance test results for pork burgers. ^a,b^ Mean values in different treatments in the same sensory attribute with different letters indicate significant difference. Sig.: significance: ** (*p* < 0.01), *** (*p* < 0.001). CON: control burgers; T1: burgers reformulated with walnut and algal oil mixture hydrogel; T2: burgers reformulated with pistachio and algal oil mixture hydrogel.

As reported in the literature, when different strategies are used to reduce the fat content of meat products, their sensory analysis must be considered. This is because fat is a fundamental ingredient in processed meat products [75] and plays an important role in multiple parameters [34]. Figure 2 presents the acceptance test results for the different treatments.

The two treatments did not significantly (*p* > 0.05) affect burger acceptability in terms of appearance, cooked odour, firmness, juiciness and fatty character, with similar values observed for all these parameters. Differences (*p* < 0.01) were observed only for flavour and overall quality parameters. Concerning flavour, the reformulation with pistachio oil did not cause any changes compared to the control elaboration, while burgers made with walnut oil were still acceptable, though they showed significantly lower values of acceptability among the three treatments. Similarly, differences were found for the overall liking values. In particular, T2 burgers had the highest overall acceptance, though there were no significant differences from the control ones, whereas T1 burgers had lower overall acceptability scores (*p* < 0.01).

According to other authors [7,23], the lower acceptability of the T1 samples could be explained by its characteristic flavour, which is not described as a typical burger flavour. Barros et al. [23] have obtained similar results to ours using wheat germ and/or algal oil emulsion hydrogels in deer burgers, finding only differences for flavour and overall quality parameters. In addition to this, Vargas-Ramella et al. [21] reported the highest overall acceptance in samples made with tiger nut oil but no significant differences among burgers from control, tiger nut and linseed treatments, while chia samples showed the lowest overall acceptability score. On the other hand, according to Dominguez et al. [76], the use of emulsion hydrogels in patties and burgers as animal fat replacers did not influence or increase consumer acceptability and some studies reporting partial or total animal fat substitution by algal oil [12], tiger nut oil [5,21], linseed oil [21] and avocado or pumpkin seed oil [7] emulsion hydrogels in burgers can confirm that. Nevertheless, taking the results into account, it is possible to affirm that all formulations were considered “acceptable” (>3.5 “acceptability limit”).

Regarding the preference test, Table 4 indicates the order of preference for treatments, from the most preferred (extreme left) to the least preferred (extreme right), in descending order of sum of scores in relation to appearance, cooked odour, firmness, juiciness, fatty character, flavour and global preference. Treatments located between the extremes are considered as “neither most nor least preferred” in relation to other treatments. Significant differences are indicated by placing treatments in descending lines with respect to a sensory attribute. For most of the attributes (appearance, firmness, fatty character and flavour), T2 samples were preferred over T1, except for cooked odour and juiciness, where no differences were observed between the three treatments.

Moreover, in line with the trend observed in the preference test, T2 burgers were also preferred over the control group for the flavour character, presenting the highest score. Confirming this, analysing the scores of global preferences, it is possible to certify that T2 burgers were the favourite ones (the ones most chosen by consumers) and that T1 burgers were the least favourite, with no significant differences from the control treatment. Therefore, Friedman’s test indicated that total preference was affected (*p* < 0.05) by the type of fat source included in the formulations (F test < F = 0.05). Previous studies reported different results from ours, such as Martins et al. [77], who reported a clear preference for the control samples (more than 80%) and Cittadini et al. [7] and Barros et al. [5], who reported no significant outcomes in the preference test. Considering the sensorial analysis data, it seems clear that the oil hydrogel emulsions used in this study for the formulation of T1 and T2 burgers could represent successful pork backfat replacers to obtain healthier products without jeopardising acceptability and consumer preference.

## 4. Conclusions

The results obtained showed how the two reformulations were able to reduce the fat amount to elaborate healthier pork burgers, following health recommendations and consumer requests. Both oil hydrogel emulsions reduced SFA and increased PUFA contents in modified pork burgers. The pistachio oil emulsion (T2) also increased MUFA amounts (mainly oleic acid) because of the high content in the oil employed. The use of algal oil in both formulations caused an increase in omega-3 content (mostly DHA), improving considerably the nutritional value of the final products which can be claimed as having a “high omega-3 content”. In addition to reducing the fat content of the burgers, the proposed treatments resulted in a significant reduction in the total energy content and an improvement in all health indices considered (n-6/n-3, PUFA/SFA, TI, AI and h/H). Moreover, the total replacement of pork fat by these hydrogel emulsions allowed the production of burgers that are considered acceptable by consumers. In fact, all the formulations showed good acceptability values, but it is important to note that, although not significantly, the T2 sample showed higher levels of acceptability in terms of flavour parameter and overall acceptability compared to the T1 and control samples, while the use of walnut oil significantly reduced these two parameters. Again, from a sensory point of view, the data for global preferences showed that consumers preferred the T2 sample over the T1 and control burgers. Certainly, more studies are needed to improve the technological properties (colour and texture) of reformulated burgers, but it is important to consider that oil mixtures may be a better strategy than the use of a single oil because of different fatty acid profiles. Therefore, based on the results obtained in the present study, it is possible to conclude that the addition of algal oil and pistachio or walnut oil hydrogel emulsions to replace animal fat in pork burgers can be an effective method to reduce fat amounts and improve nutritional characteristics compared to the traditional product, without affecting consumers’ perceptions and the sensory characteristics of the products. Finally, further evaluation of pork burgers produced with algal oil and pistachio or walnut oil hydrogel emulsions during storage will be useful to characterize quality changes (such as microbial growth) and determine shelf life.

## Figures and Tables

**Table 1:** Fatty acid composition (g/100 g fat) of fat sources used in the burger formulation.

Fatty Acids	Treatments				SEM	Sig.
	Pork Backfat	Walnut Oil	Pistachio Oil	Algal Oil		
C12:0	0.10 ^b^	0.00 ^a^	0.01 ^a^	0.72 ^c^	0.091	***
C14:0	1.22 ^b^	0.02 ^a^	0.09 ^a^	8.72 ^c^	1.093	***
C14:1n-5	0.01 ^a^	0.00 ^a^	0.00 ^a^	0.17 ^b^	0.022	***
C15:0	0.06 ^b^	0.01 ^a^	0.01 ^a^	0.53 ^c^	0.066	***
C16:0	23.71 ^d^	6.31 ^a^	11.01 ^b^	15.17 ^c^	1.936	***
C16:1n-7	1.52 ^b^	0.08 ^a^	1.01 ^b^	7.93 ^c^	0.938	***
C18:0	13.00 ^d^	2.44 ^c^	1.37 ^b^	0.52 ^a^	1.524	***
9t-C18:1	0.17 ^c^	0.03 ^a^	0.02 ^a^	0.09 ^b^	0.018	***
11t-C18:1	0.20 ^d^	0.10 ^b^	0.14 ^c^	0.08 ^a^	0.014	***
C18:1n-9	35.00 ^c^	18.68 ^b^	52.17 ^d^	0.15 ^a^	5.814	***
C18:1n-7	2.22 ^b^	0.88 ^a^	2.45 ^b^	7.37 ^c^	0.748	***
C18:2n-6	14.46 ^b^	58.43 ^d^	28.64 ^c^	0.09 ^a^	6.508	***
C18:3n-6	0.02 ^b^	0.00 ^a^	0.00 ^a^	0.11 ^c^	0.014	***
C18:3n-3	0.76 ^b^	12.82 ^c^	0.46 ^b^	0.01 ^a^	1.623	***
9c,11t-C18:2 (CLA)	0.13 ^d^	0.07 ^c^	0.05 ^b^	0.00 ^a^	0.014	***
C20:0	0.23 ^d^	0.11 ^b^	0.17 ^c^	0.05 ^a^	0.020	***
C20:1n-9	0.81 ^d^	0.20 ^b^	0.33 ^c^	0.02 ^a^	0.088	***
C20:2n-6	0.67 ^d^	0.04 ^c^	0.03 ^b^	0.00 ^a^	0.084	***
C20:3n-6	0.15 ^c^	0.00 ^a^	0.00 ^a^	0.12 ^b^	0.021	***
C20:4n-6	0.22 ^b^	0.00 ^a^	0.00 ^a^	0.24 ^b^	0.035	***
C20:3n-3	0.13 ^b^	0.00 ^a^	0.00 ^a^	0.00 ^a^	0.017	***
C22:0	0.03 ^b^	0.05 ^c^	0.12 ^d^	0.00 ^a^	0.013	***
C20:5n-3 (EPA)	0.03 ^a^	0.00 ^a^	0.00 ^a^	1.42 ^b^	0.186	***
C24:0	0.04 ^a^	0.05 ^b^	0.10 ^c^	0.16 ^d^	0.013	***
C22:5n-3 (DPA)	0.17 ^b^	0.00 ^a^	0.00 ^a^	0.45 ^c^	0.055	***
C22:6n-3 (DHA)	0.06 ^a^	0.00 ^a^	0.07 ^a^	44.99 ^b^	5.890	***
SFA	38.94 ^d^	9.13 ^a^	13.05 ^b^	26.19 ^c^	3.547	***
MUFA	39.97 ^c^	20.02 ^b^	56.18 ^d^	15.85 ^a^	4.887	***
PUFA	16.81 ^a^	71.35 ^d^	29.25 ^b^	47.43 ^c^	6.219	***
n-3	1.14 ^a^	12.82 ^b^	0.53 ^a^	46.87 ^c^	5.709	***
n-6	15.53 ^b^	58.47 ^d^	28.67 ^c^	0.56 ^a^	6.432	***
LC n-3	0.26 ^a^	0.00 ^a^	0.07 ^a^	46.87 ^b^	6.127	***

Results in the same row with the same superscripts indicates no significant differences. Sig.: significance: *** (*p* < 0.001). LC n-3: long-chain omega-3 (sum of EPA, DPA and DHA).

**Table 2:** Proximate composition, lipid oxidation and physicochemical properties of pork burgers.

Parameters	Treatments			SEM	Sig.
	CON	T1	T2		
Chemical composition (g/100 g)					
Moisture	70.45 ^a^	74.26 ^b^	74.56 ^b^	0.296	***
Fat	10.03 ^b^	5.92 ^a^	5.64 ^a^	0.319	***
Protein	17.34	16.83	16.80	0.111	ns
Ash	1.90 ^a^	2.24 ^b^	2.23 ^b^	0.026	***
Energy content (Kcal/100 g)	159.69 ^b^	120.60 ^a^	117.98 ^a^	2.981	***
Energy reduction (%)	0.00 ^a^	24.46 ^b^	26.13 ^b^	1.841	***
Fat reduction (%)	0.00 ^a^	41.07 ^b^	43.14 ^b^	3.077	***
Colour parameters					
L*	55.46 ^a^	64.06 ^c^	61.70 ^b^	0.650	***
a*	11.60	10.58	11.11	0.216	ns
b*	18.87 ^a^	20.49 ^b^	19.99 ^a b^	0.279	*
pH	5.67	5.63	5.61	0.018	ns
Cooking loss (%)	26.41	25.47	25.03	0.299	ns
Texture parameters					
Hardness (N)	54.29 ^a^	68.67 ^b^	68.01 ^b^	1.257	***
Springiness (mm)	0.76	0.75	0.75	0.004	ns
Cohesiveness	0.57 ^a^	0.59 ^b^	0.59 ^b^	0.004	*
Gumminess (N)	30.83 ^a^	40.05 ^b^	39.66 ^b^	0.812	***
Chewiness (N·mm)	23.09 ^a^	30.45 ^b^	29.59 ^b^	0.658	***

Results in the same row with the same superscripts indicates no significant differences. Sig.: significance: * (*p* < 0.05); *** (*p* < 0.001); ns: not significant. CON: control burgers; T1: burgers reformulated with walnut and algal oil mixture hydrogel; T2: burgers reformulated with pistachio and algal oil mixture hydrogel.

**Table 3:** Fatty acid composition (expressed as g/100 g of fat) of pork burgers.

Fatty Acids	Treatments			SEM	Sig.
	CON	T1	T2		
C12:0	0.09 ^b^	0.07 ^a^	0.07 ^a^	0.002	***
C14:0	1.15 ^b^	0.83 ^a^	0.82 ^a^	0.027	***
C16:0	22.47 ^c^	14.29 ^a^	15.54 ^b^	0.578	***
C16:1n-7	1.69 ^b^	1.52 ^a^	1.86 ^c^	0.032	***
C17:0	0.34 ^b^	0.16 ^a^	0.16 ^a^	0.013	***
C18:0	11.70 ^c^	5.99 ^b^	5.00 ^a^	0.454	***
9t-C18:1	0.07 ^b^	0.04 ^a^	0.05 ^a b^	0.006	*
11t-C18:1	0.09	0.07	0.05	0.008	ns
C18:1n-9	33.10 ^b^	26.03 ^a^	40.30 ^c^	0.955	***
C18:1n-7	2.72 ^a^	2.58 ^a^	3.35 ^b^	0.065	***
C18:2n-6	13.44 ^a^	31.15 ^c^	19.25 ^b^	1.144	***
C18:3n-3	0.66 ^a^	5.78 ^b^	0.48 ^a^	0.375	***
9c,11t-C18:2 (CLA)	0.12 ^c^	0.10 ^b^	0.09 ^a^	0.002	***
C20:0	0.19 ^c^	0.13 ^a^	0.15 ^b^	0.004	***
C20:1n-9	0.72 ^c^	0.42 ^a^	0.45 ^b^	0.021	***
C20:2n-6	0.59 ^c^	0.28 ^b^	0.25 ^a^	0.023	***
C20:3n-6	0.17 ^b^	0.14 ^a^	0.15 ^a^	0.002	***
C20:4n-6	0.58 ^a^	0.75 ^b^	0.75 ^b^	0.021	***
C20:3n-3	0.11 ^b^	0.04 ^a^	0.04 ^a^	0.005	***
C20:5n-3 (EPA)	0.03 ^a^	0.08 ^b^	0.09 ^c^	0.004	***
C22:5n-3 (DPA)	0.20 ^a^	0.22 ^b^	0.21 ^a b^	0.003	*
C22:6n-3 (DHA)	0.06 ^a^	1.29 ^b^	1.43 ^c^	0.094	***
SFA	36.21 ^b^	21.73 ^a^	22.09 ^a^	1.062	***
MUFA	38.43 ^b^	30.70 ^a^	46.10 ^c^	1.044	***
PUFA	15.99 ^a^	39.85 ^c^	22.77 ^b^	1.555	***
n-3	1.06 ^a^	7.41 ^c^	2.25 ^b^	0.422	***
n-6	14.81 ^a^	32.34 ^c^	20.43 ^b^	1.139	***
LC n-3	0.29 ^a^	1.58 ^b^	1.73 ^c^	0.099	***
n-6/n-3	13.93 ^c^	4.38 ^a^	9.10 ^b^	0.589	***
PUFA/SFA	0.44 ^a^	1.87 ^c^	1.04 ^b^	0.093	***
TI	1.18 ^c^	0.39 ^a^	0.53 ^b^	0.052	***
AI	0.50 ^c^	0.25 ^a^	0.27 ^b^	0.017	***
h/H	2.15 ^a^	4.44 ^c^	3.94 ^b^	0.154	***

Results in the same row with the same superscripts indicates no significant differences; Sig.: significance: * (*p* < 0.05), *** (*p* < 0.001); ns: not significant; CON: control burgers; T1: burgers reformulated with walnut and algal oil mixture hydrogel; T2: burgers reformulated with pistachio and algal oil mixture hydrogel.

**Table 4:** Preference test of pork burgers.

	Most Favourite Sample		Least Favourite Sample
Appearance	CON(88)	T2 (80)	T1(60)
	
		
Cooked odour	T2 (85)	CON(73)	T1(70)
		
Firmness	T2 (91)	CON(78)	T1(59)
	
		
Juiciness	T2 (86)	CON(74)	T1(68)
		
Fatty character	T2 (92)	CON(75)	
		CON(75)	T1(61)
	
Flavour	T2 (94)		
		CON(75)	T1(59)
	
Global preference	T2 (97)		
		CON(71)	T1(60)
	

Samples in the same row did not have significant differences (*p* > 0.05). Numbers in brackets are the sum of scores. CON: control burgers; T1: burgers reformulated with walnut and algal oil mixture hydrogel; T2: burgers reformulated with pistachio and algal oil mixture hydrogel.

## Data Availability

The data presented in this study are available on request from the corresponding author. The data are not publicly available due to ethical reasons.
